# Distribution of the Cannabinoid Receptor Type 1 in the Brain of the Genetically Audiogenic Seizure-Prone Hamster GASH/Sal

**DOI:** 10.3389/fnbeh.2021.613798

**Published:** 2021-03-24

**Authors:** Alejando Fuerte-Hortigón, Jaime Gonçalves, Laura Zeballos, Rubén Masa, Ricardo Gómez-Nieto, Dolores E. López

**Affiliations:** ^1^Institute of Neurosciences of Castilla y León, University of Salamanca, Salamanca, Spain; ^2^Department of Neurology, Virgen Macarena Hospital, Sevilla, Spain; ^3^Institute of Biomedical Research of Salamanca, Salamanca, Spain; ^4^Department of Cell Biology and Pathology, University of Salamanca, Salamanca, Spain

**Keywords:** cannabinoid receptors, GASH/Sal, epilepsy, gene expression, immunohistochemistry

## Abstract

The endocannabinoid system modulates epileptic seizures by regulating neuronal excitability. It has become clear that agonist activation of central type I cannabinoid receptors (CB1R) reduces epileptogenesis in pre-clinical animal models of epilepsy. The audiogenic seizure-prone hamster GASH/Sal is a reliable experimental model of generalized tonic-clonic seizures in response to intense sound stimulation. However, no studies hitherto had investigated CB1R in the GASH/Sal. Although the distribution of CB1R has been extensively studied in mammalian brains, their distribution in the Syrian golden hamster brain also remains unknown. The objective of this research is to determine by immunohistochemistry the differential distribution of CB1R in the brains of GASH/Sal animals under seizure-free conditions, by comparing the results with wild-type Syrian hamsters as controls. CB1R in the GASH/Sal showed a wide distribution in many nuclei of the central nervous system. These patterns of CB1R-immunolabeling are practically identical between the GASH/Sal model and control animals, varying in the intensity of immunostaining in certain regions, being slightly weaker in the GASH/Sal than in the control, mainly in brain regions associated with epileptic networks. The RT-qPCR analysis confirms these results. In summary, our study provides an anatomical basis for further investigating CB1R in acute and kindling audiogenic seizure protocols in the GASH/Sal model as well as exploring CB1R activation via exogenously administered cannabinoid compounds.

## Introduction

The endocannabinoid system consists of specific cannabinoid receptors, their endogenous ligands and the enzymatic systems of their biosynthesis and degradation (Svízenská et al., 2008). This system is widespread in the central nervous system and is involved in the regulation of the following processes: neurogenesis, memory, appetite, metabolism, stress, emotions, immune response, anxiety, analgesia, thermoregulation, sleep, perception, motor coordination, behavior, and reproduction (Chaperon and Thiébot, 1999; Viveros et al., 2005; Fernández-Ruiz et al., 2007; Crowe et al., 2014; Hillard, 2014; Soria-Gómez et al., 2014; Gatta-Cherifi and Cota, 2015; Lu and Potter, 2017; Robertson et al., 2017).

Endocannabinoids inhibit retrograde release of some neurotransmitters such as γ-aminobutyric acid, glutamate and serotonin (Pazos et al., 2005), since they regulate the aperture/closing of ion channels (Childers and Breivogel, 1998; Qian et al., 2017) in both excitatory (glutamatergic) and inhibitory (GABAergic) synapses (Julian et al., 2003), in response to an increase in the intracellular Ca^2+^ concentration (Ohno-Shosaku and Kano, 2014; Kendall and Yudowski, 2016). These regulatory effects are primarily mediated by two G-protein-coupled receptors: cannabinoid receptor type 1 (CB1R) and cannabinoid receptor type 2 (CB2R) (Marcu and Schechter, 2016; Lu and Potter, 2017). The functions of endocannabinoids in the central nervous system are carried out by activation of CB1R, while CB2R plays a much more limited role. CB1Rs are expressed on presynaptic terminals of neurons of the central nervous system (Pazos et al., 2005; Kendall and Yudowski, 2016). However, this does not preclude the existence of CB1Rs at postsynaptic sites, as functional studies demonstrate self-inhibition in neocortical neurons by endocannabinoids (Maroso et al., 2016). In addition, a high proportion of CB1Rs, at steady state, is localized to somatodendritic endosomes (Thibault et al., 2013). These receptors are also present in the astrocytes, oligodendrocytes and in the cerebral vasculature. Specifically, CB1Rs are highly expressed in brain areas responsible for mood regulation, motor co-ordination, cognition and pain such as the hippocampus, olfactory regions, caudate putamen, accumbens nucleus, substantia nigra, globus pallidus, periaqueductal gray matter, dorsal horn of the medulla, cingulate gyrus, neocortex, amygdala, hypothalamus, and solitary nucleus (Tsou et al., 1998; Svízenská et al., 2008; Hu and Mackie, 2015).

CB1 and CB2 receptors can be activated by exogenous cannabinoids, producing the biological effects of endocannabinoids. Cannabis plants contain more than a 100 terpenophenolic compounds that have been called cannabinoids (Gould, 2015), the two most abundant being Δ9-THC (Δ9-tetrahydrocannabinol) and CBD (cannabidiol).

Currently, there has been growing interest in the use of exogenous cannabinoid compounds for the treatment of a variety of neurological diseases, including epilepsy (Sulak et al., 2017). CBD has been approved in some countries for the treatment of drug-resistant epileptic syndromes (Dravet and Lennox–Gastaut Syndromes) (Devinsky et al., 2014; Thiele et al., 2018). It is estimated that 25–30% of epileptic patients suffer from intractable seizures that cannot be controlled by antiepileptic medications (O'Connell et al., 2017) and they often require invasive treatments such as neurostimulation or surgical resection (Reddy and Golub, 2016). In addition, the development of a single drug which could control seizures would reduce the probability of developing toxic effects (Wilby et al., 2005). It has been demonstrated that there is a pathophysiological reorganization of the endocannabinoid system (Blair et al., 2015; Katona, 2015) and an activation of CB1R as a protective mechanism against excitotoxicity in epileptic patients (Lupica et al., 2017). Both direct (the use of CB1R agonists) and indirect approaches (inhibition of endocannabinoid catabolism) reduce epileptogenesis in animal models (Rosenberg et al., 2017). Endocannabinoid signaling mediated through presynaptic CB1R reduces both glutamate and GABA release (Kathmann et al., 1999; Misner and Sullivan, 1999; Hájos et al., 2000), and therefore is a potent regulator of neuronal excitability. CB1R agonists have been widely studied for anti-seizure effects across an array of models of seizures; this has been reviewed extensively elsewhere (Wallace et al., 2001; Skaper and Di Marzo, 2012; Cristino et al., 2020). The study of the endocannabinoid signaling pathway, its physiological action and distribution are key for the development of more treatments based on exogenous cannabinoid compounds.

We therefore set out to study the distribution of the main brain cannabinoid receptor, CB1R, in the GASH/Sal epilepsy model under seizure-free conditions, by comparing the results with wild-type Syrian hamsters, since these elements can become pharmacological targets for the treatment of epilepsy, where an alteration of this system is postulated.

## Materials and Methods

### Animals

Fourteen GASH/Sal and 13 male Syrian hamsters 4 months of age were obtained from the Animal Facility of the University of Salamanca (USAL, Spain) and Janvier Labs (Le Genest-Saint-Isle, France), respectively, to be used in this experiment. Male hamsters were selected in order to remove potentially confounding hormonal processes intrinsic to female metabolism. Animals were maintained under normal conditions of lighting (12 h light/dark cycle) and temperature (22 ± 1°C) in an acoustically controlled environment, and with free access to water and food.

All the procedures and experimental protocols were performed in accordance with the guidelines of the European Community's Council Directive (2010/63/EU) and approved by the Bioethics Committee of the University of Salamanca (approval number 380).

### Quantitative Reverse Transcription PCR (RT-qPCR)

The primers were designed for *Mesocricetus auratus*. Gene sequences were obtained from the Ensembl Genome Browser database (http://www.ensembl.org/index.html) and the primers were designed aligned in different exons using the Primer3 software (http://bioinfo.ut.ee/primer3-0.4.0/primer3/) (Table 1). The primers were synthesized by Thermo Fisher Custom Primers (Invitrogen - Thermo Fisher).

**Table 1:** Primers used for RT-qPCRs.

**Gen targetet**	**ID transcript Ensembl *Mesocricetus auratus*^**a**^**	**Primer forward**	**Primer reverse**	**Size of products**	**E^***b***^**
*Cb1r*	*ENSMAUG00000014040*	TGTTGACTTCCATGTGTTCCA	GGTCTGGTGACGATCCTCTT	171	1.15
*Actb*	*ENSMAUG00000008763*	AGCCATGTACGTAGCCATCC	ACCCTCATAGATGGGCACAG	105	2.03

*List of primers used for RT-qPCRs, indicating the location of each primer in the corresponding Ensembl sequences of the Syrian hamster (a). qPCR primer efficiency (E^b^) was calculated according to the following equation: E = 10^(−1/slope)^*.

A total of 12 animals (6 control hamsters and 6 GASH/Sal) were deeply anesthetized by isoflurane inhalation and once areflexia was verified, were decapitated. Different brain structures were removed for gene expression studies: brain stem, cerebellum, inferior colliculus, hippocampus and cortex. All tissues harvested were put into storage at −80°C until use. The RT-qPCR approach was identical to that used previously by our group (e.g., Damasceno et al., 2020; Sánchez-Benito et al., 2020). RNA from samples was extracted in accordance with the protocol of TRIzol^™^ Reagent (#15596026, Invitrogen). Total RNA concentration was quantified using the NanoPhotometer^®^/spectrophotometer (Implen, Munich, Germany), taking into account the absorption ratios 260/280 nm and 260/230 nm, and RNA integrity was checked by electrophoresis in agarose gel (1.5%). Genomic DNA was degraded using the Ambion^™^ DNase I (RNase free) (Thermo Fisher Scientific) following the supplier's instructions.

Complementary DNA (cDNA) was synthesized from 800 ng of total RNA using the ImProm-IITM Reverse Transcription System Kit (Promega Corporation, Madison, SWI, USA). The relative quantification of the transcripts was performed on ABI Prism 7000 (Applied Biosystems) using the SYBR Green Master Mix (#4309155, Applied Biosystems). Initially, a serial dilution curve was made to verify the efficiency of the primers of the target and reference genes.

The quantitative reverse transcription real time PCR was conducted using the SYBER Green method. Each reaction contained 7 μL of SYBR, 30 ng of total cDNA, 0.8 μL of each primer (10 μM), and MiliQ water free of DNase and RNase up to 20 μL. The cycling conditions were in accordance with the protocol of the intercalating agent used. RT-qPCR experiments were performed in replicates of four to six samples and conducted in triplicate for the gene product examined, and β-actin (*Actb*) was used as a negative control. Following the removal of outliers (Burns et al., 2005), raw data was used to determine the PCR amplification efficiency (E). The relative gene expression value for each transcript was calculated according to the formula 2^−(1Ct “condition 1”−1Ct “condition 2”)^, where “condition 1” corresponds to the experimental sample, “condition 2” corresponds to the sample from the control animal, and 1Ct of each “condition” is Ct_“experimental gene”_ – Ct_“endogenous gene”_ (Schmittgen and Livak, 2008). The relative mRNA of the groups was evaluated using an unpaired *t*-test. The analyses were performed using GraphPad Prism 7. *p* < 0.05 was considered as statistically significant. All quantitative data were expressed as mean value ± standard error of the mean (SEM). Asterisks indicate significant differences between experimental groups (“^*^” = *p*-value < 0.05; “^**^” = *p*-value < 0.01; “^***^” = *p*-value < 0.001).

### Brain Tissue Processing and Immunostaining

Brain tissue used for immunohistochemistry (3 control and 4 GASH/Sal hamsters) was processed in accordance with the routine protocols used in the laboratory (Sánchez-Benito et al., 2020). Briefly, after injection of a lethal dose of sodium pentobarbital (60 mg.kg^−1^) and the subsequent perfusion through the heart with 4% paraformaldehyde in 0.1 M phosphate buffer saline (PBS), brains were removed from the skull, cryoprotected by immersion in 30% sucrose, and coronal sections were cut with a freezing sliding microtome at 40 μm thickness. Serial sections were collected in PBS and divided into a series of 6 and placed in wells containing 0.1 M-phosphate buffer.

The CB1R was visualized following the indirect method of immunohistochemical staining described by Sánchez-Benito et al. (2020). A primary polyclonal antibody anti-CB1R obtained in rabbit (CB1-Rb-Af380, Frontier Institute, Hokkaido, Japan) which binds to the C-terminal (NM007726) of the mouse protein CB1R was used since there was no primary antibody Anti-CB1R available specific for GASH/Sal hamster. Its reactivity in mice was tested by immunoblot following the manufacturer's instructions. The CB1 protein sequence corresponding to the *cnr1* gene was retrieved from the UNIPROT protein database (https://www.uniprot.org/), and then analyzed using the EBI-Clustal Omega program (http://www.ebi.ac.uk/Tools/msa/clustalo/) (Sievers and Higgins, 2018). The sequence is highly conserved between the CB1R in the hamster and mouse (Supplementary Material 1). Washes were made in Tris-buffered saline (TBS), pH 7.4 and dilutions of antisera in TBS containing 0.2% Triton X-100 (# T9284; Sigma).

For light microscopy analysis, free-floating sections were blocked for 1 h with 5% normal goat serum (#S-1000, Vector Labs.) in TBS-Tx and were incubated with primary antibodies at 1:250 dilution for 72 h at 4°C. Sections were then washed and followed an incubation with the biotinylated secondary antibodies, goat anti-rabbit (#BEA-1000, Vector Labs.), at 1:200 dilution for 2 h. After removal of secondary antisera, the visualization of epitope-antibody interactions was developed with the avidin-biotin peroxidase complex procedure (#PK-4000, Vectastain, Vector Labs.), and diaminobenzidine histochemistry for peroxidase (DAB Kit, #SK-4100, Vector Labs.). All sections were mounted onto slides, (ordered rostro-caudally), dehydrated and coverslipped with Entellan^®^ Neu (#107961, Merck).

To visualize the morphological features of immunostained cells, we used brain embedded in paraffin wax (2 control and 2 GASH/Sal hamsters) before cutting into coronal sections of 6 μm thickness, according to the protocols routinely used in our laboratory (Sánchez-Benito et al., 2020). Then, sections were mounted onto slides and followed the immunohistological staining procedure to visualize the CB1R protein at optical and confocal laser scanning microscopes. In order to identify the possible glial nature of the immunolabeled small cells, a GFAP marker was used, performing a double fluorescent labeling on the 6 μm brain sections, incubating the horizontally arranged slides in a humid chamber.

After deparaffinization and rehydration, endogenous peroxidase activity was blocked with 2.5% horse serum (#S-2000-20, Vector Labs.) and incubation with primary antibodies (rabbit anti CB1R and mouse anti GFAP) was carried out. Subsequently, the sections were rinsed extensively and reacted for 30 min with secondary antibody, VectaFluor^™^ Duet Reagent [#DK-8818, DyLight^®^ 488 Anti-Rabbit IgG and DyLight^®^ 594 Anti-Mouse IgG cocktail (anti-rabbit Ig in green, anti-mouse in red)] made in horse. Finally, sections were coverslipped with VECTASHIELD^®^ mounting medium for preserving fluorescence, containing the DAPI counterstain (4,6-diamidino-2-phenylindole, #H-1200, Vector Labs.). Additionally, alternative sections were counterstained with Nissl stain, dehydrated and cover slipped with Entellan^®^ Neu, #107961, Merck. A list of the antibodies used is shown in Table 2.

**Table 2:** List of antibodies used.

**Antigen**	**Primary AB**	**Dilution**	**Reference**	**Secondary AB**	**Dilution**	**Reference**	**Objective**
CB1R	Rabbit anti CB1R	**1/250**	**CB1-Rb-Af380-*****Fr***	Biotinilated goat anti rabbit-*Vec*	1/200	BEA-1000-*Vec*	Light Microscopy
				DyLight^®^ 488anti-rabitt-*Vec*	1/200	DK-8818-*Vec*	Confocal Microscopy
GFAP	mouse anti GFAP	**1/2,000**	**G6171-*****Sig***	DyLight^®^ 594anti-mouse-*Vec*	1/200		

*Comercials*.

*Fr- Frontier Institute, Hokkaido, Japón*.

*Sig-Sigma-Aldrich,Taufkirchen, Germany*.

*Vec- Vector Laboratoires, Burlingame, CA, USA*.

### Immunoblotting

Cerebellum samples corresponding to age- and sex-matched animals (two GASH/Sal and two control hamsters, males with 4 months of age) were used to verify that the primary antibody against CB1R specifically detects its antigen in a western blot experiment. In Brief, the cerebellum samples were homogenized with IKA T10 Basic Ultra Turrax homogenizer (IKA, Germany) in ice-cold RIPA buffer containing protease inhibitors (Cell Signaling Technologies, USA). Supernatants of the homogenates were collected after centrifugation at 14,000 rpm (Centrifuge 5417R, Eppendorf, Germany) for 15 min, and the protein concentration was determined using the Lowry method. The Samples (150 μg) were separated by gel electrophoresis, using 10% TGX precast gels (Bio-Rad, United States), and electroblotted onto a PVDF membrane (Merck, Germany), which was incubated overnight with the polyclonal antibody anti-CB1R (dilution 1:1,000) at 4°C. The membrane was then immunoreacted for 1 h with the HRP-linked secondary antibody (anti-rabbit IgG) at 1:15,000 dilution (Cell Signaling Technologies, USA). Finally, the immunoreaction was visualized with the ImageQuant RT ECL detection system (GE Healthcare, USA).

### Observation and Study of Histological Samples

Sections were observed using a Leica LB30T microscope equipped with a digital camera (Olympus DT70). The photographs were processed with minor modifications in contrast using Adobe Photoshop CS2. Figures were assembled using Canvas Draw 2. “A Stereotaxic Atlas of the Golden Hamster Brain” (Morin and Wood, 2001) was used as a reference to classify histological sections rostro-caudally arranged. In all immunohistochemical experiments, omission of primary antibody resulted in absence of staining of the preparations. The sections processed for immunofluorescence were studied on a Leica Stellaris confocal laser coupled to a Leica Zeiss Axio Observer DMI8 microscope, using the appropriate filters for DyLight^®^ 594 (red), DyLight^®^ 488 (green) and DAPI (violet) fluorochromes. These three fluorochromes were detected sequentially, stack by stack, with the acousto-optical beam splitter as tunable dichroic filter system, using the laser spectral lines 488, 594, and 405 nm, respectively. The objectives used were x40 and oil immersion x63/numerical aperture 1.40, pinhole 1 Airy unit, as well as several electronic zoom factors. To determine the distribution of the immunolabeled terminals, series of 10–15 confocal images were obtained to generate a maximal-intensity z projection of stacks. Colocalization of the fluorochromes DyLight^®^ 488 and DyLight^®^ 594 within positive terminals was always verified in the orthogonal view (=xy, xz, yz planes, for z stacks series). A sequence of 15 serial pictures from different viewpoints was created to produce a three-dimensional (3D) animation and the movie document generated from the image stacks were stored at 30 frames per second as a Windows Media Video file.

## Results

### Distribution of CB1 Receptors in the Brain of GASH/Sal

**Figure 1:**
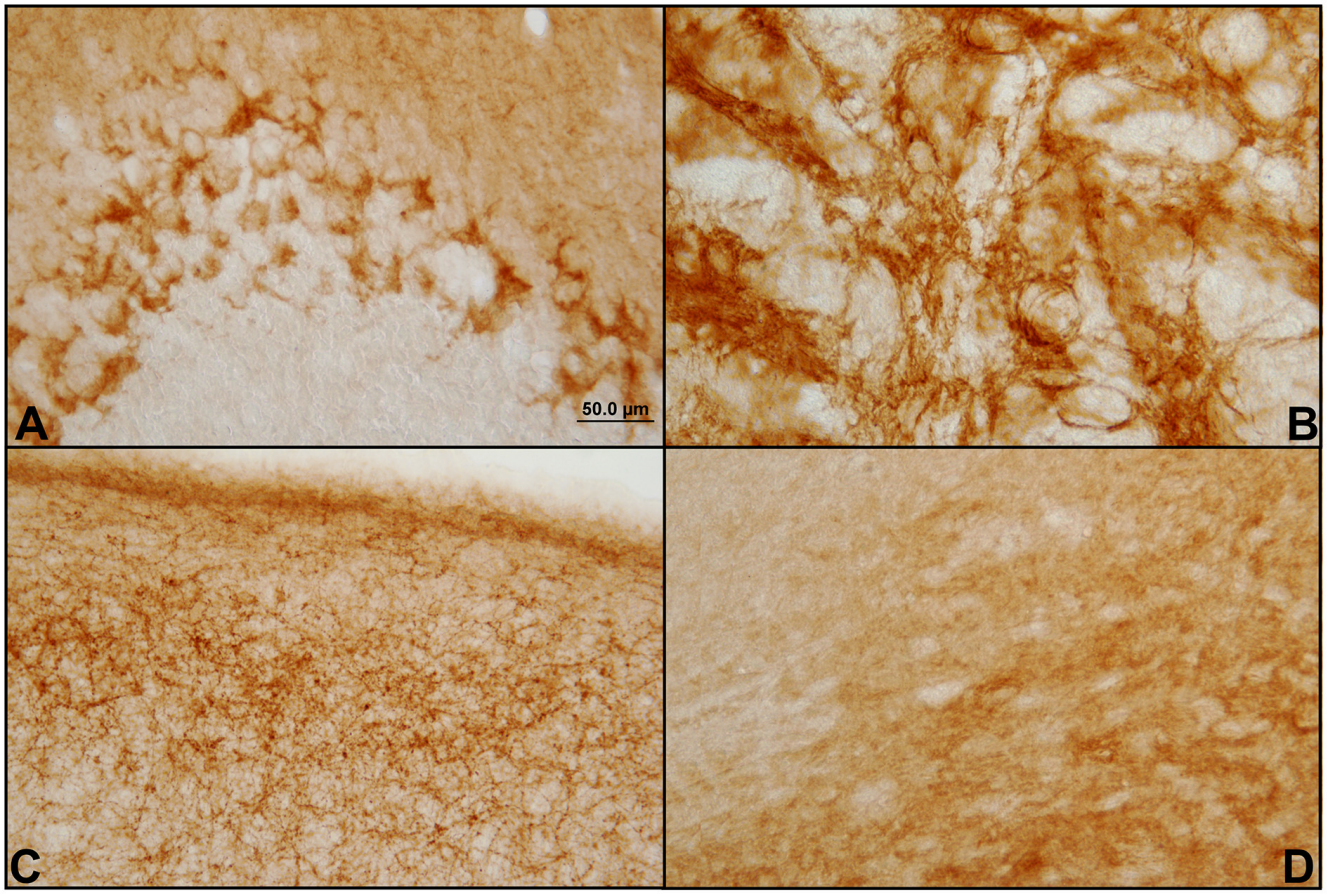
Different patterns of immunostaining of CB1 receptors in the hamster GASH/Sal brain. Immunostaining of CB1 receptors in the brain of GASH/Sal in which a representation of the different staining patterns shown in coronal sections of different brain areas. **(A)** Dot-like staining surrounding the Purkinje cells bodies of the cerebellum; **(B)** Network of fibers (reticular staining) in the globus pallidus nucleus; **(C)** Plexiform staining in the primary motor cortex; **(D)** Diffuse staining in the substantia nigra.

The antibody used in a dilution of 1:250 provides immunoreactivity in the central nervous system of the *Mesocricetus auratus*, both in the GASH/Sal line and in Syrian control hamsters. None of the performed controls yielded false positives. Significant immunolabeling was observed in numerous areas of the GASH/Sal brain. Different types of immunolabeling patterns were distinguished, based on the histology of each area, as well as differences in the immunostaining intensity (Figure 1).

We found strong and intense CB1R-immunolabeling in the following brain areas: cerebellum, substantia nigra, motor cortex, hippocampus, endopiriform nucleus, subtalamic nuclei, globus pallidus and olfactory bulb. Intense immunoreactivity was also found in the visual, somatosensory, peripheral, and auditory and entorhinal cortices. In a more subtle and diffuse way, CB1R-immunolabeling was found in a high number of brain areas such as: periaqueductal gray matter, caudate-putamen, solitary tract, terminal stria, lateral septum, parabrachial nucleus, amygdala, lateral hypothalamus, arcuate nucleus, cuneiform nucleus, and in the insular cortex.

**Figure 2:**
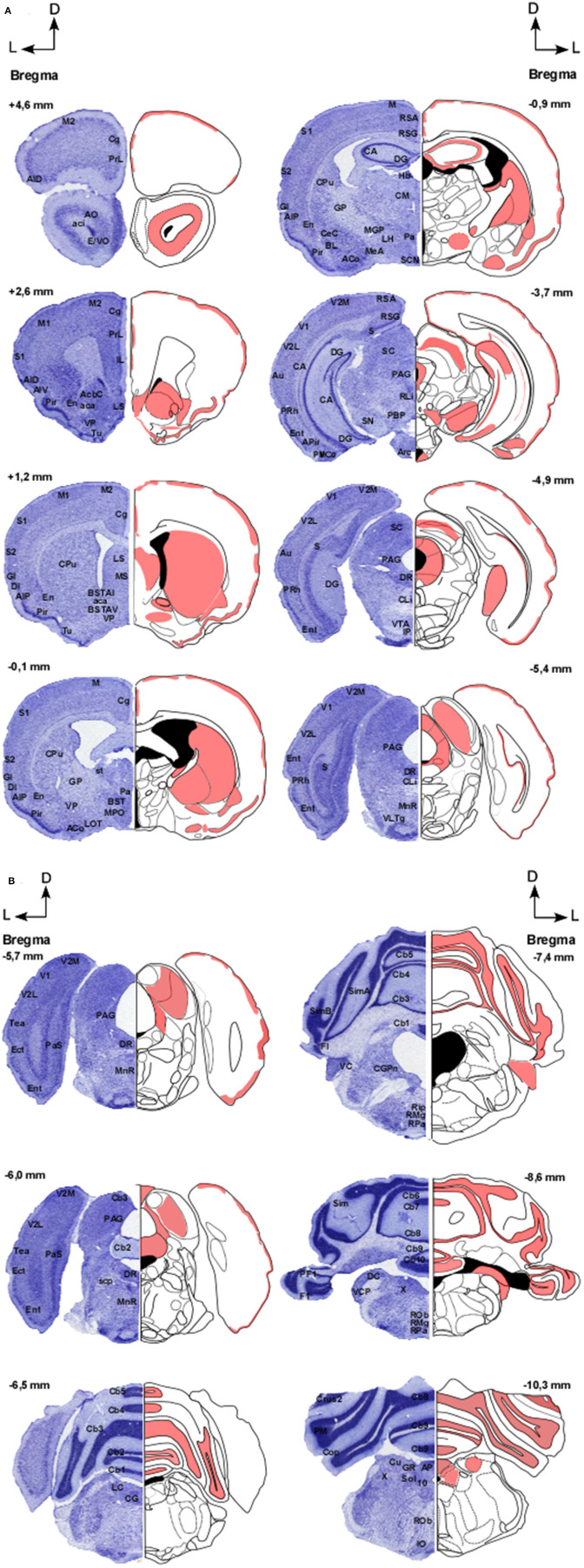
**(A,B)** Distribution pattern of CB1 receptors in the brain of GASH/Sal. Schemes showing the coronal sections of the brain at different rostro-caudal levels (referenced with respect to Bregma), according to a stereotaxic atlas of the golden hamster brain. The distribution of CB1 receptors in the GASH/Sal is shown in red. Each coronal section includes a semi-section contrasted with Nissl staining as a cytoarchitectural reference of the different nuclei. 10, Dorsal motor nucleus of the vagus; aca, Anterior commissure, anterior part; AcbC, Accumbens nucleus, core; aci, Anterior commissure, intrabulbar part; ACo, Anterior cortical amygdaloid nucleus; AID, Agranular insular cortex, dorsal part; AIP, Agranular insular cortex, posterior part; AIV, Agranular insular cortex, ventral part; AO, Anterior olfactory nucleus; AP, Area postrema; APir, Amygdalopiriform transition area; Arc, Arcuate hypothalamic nucleus; Au, Primary auditory cortex; BL, Basolateral amygdaloid nucleus; BST, Bed nucleus of stria terminalis; BSTAI, Bed nucleus of stria terminalis, anteromediate part; BSTAV, Bed nucleus of stria terminalis, anteroventral part; CA, Hippocampus; Cb1-10, Cerebellar lobule 1–10; CeC, Central amygdaloid nucleus; Cg, Cingulate cortex; CG, Central gray; CGPn, Central gray of the pons; CLi, Caudal linear nucleus of the raphe; CM, Central medial thalamic nucleus; Cop, Copula of the pyramis; CPu, Caudate putamen; Crus1-2, Crus 1-2 of the ansioform lobule; Cu, Cuneate nucleus; DC, Dorsal cochlear nucleus; DG, Dentate gyrus; DI, Dysgranular insular cortex; DR, Dorsal raphe nucleus; E/VO, Olfactory ventricle; Ect, Ectorhinal cortex; En, Endopiriform nucleus; Ent, Entorhinal cortex; F1, Flocculus; GI, Granular insular cortex; GP, Globus pallidus; GR, Gracile nucleus; Hb, Habenula nuclei; IL, Infralimbic cortex; IO, Inferior olive; IP, Interpeduncular nucleus; LC Locus coeruleus; LH, Lateral hypothalamic area; LOT, Nucleus of the lateral olfactory tract; LS, Lateral septal nucleus; M, Motor cortex; M1, Primary motor cortex; MS, Medial septal nucleus; M2, Secondary motor cortex; Mea, Medial amygdaloid nucleus; MGP, Medial globus pallidus; MnR, Median Raphe nucleus; MPO, Medial preoptic area; Pa, Paraventricular hypothalamic nucleus; PAG, Periaqueductal graymatter; Pas, Parasubiculum; PBP, Parabrachial pigmented nucleus; PF1, Paraflocculus; Pir, Piriform cortex; Pm, Paramedian lobule; PMCo, Posteromedial cortical amygdaloid nucleus; PRh, Perirhinal cortex; PrL, Prelimbic cortex; Rip, Raphe interpositus nucleus; RLi, Rostral linear nucleus of the raphe; RMg, Raphe magnus nucleus; Rob, Raphe obscurus nucleus; Rpa, Raphe pallidus nucleus; RSA, Restroplenial agranular cortex; RSG, Restrosplenial granular cortex; S, Subiculum; S1, Primary somatosensory cortex; S2, Secondary somatosensory cortex; SC, Superior colliculus; SCN, Suprachiasmatic nucleus; SimA-B, Simple lobule A-B; SN, Substantia nigra; Sol, Nucleus of the solitary tract; st, Stria terminalis; scp, Superior cerebellar penduncle; Tea, Temporal Association Cortex; Tu, Olfactory tubercle; V1, Primary visual cortex; V2L, Secondary visual cortex, lateral part; V2M, Secondary visual cortex, medial part; VC, Ventral cochlear nucleus; VCP, ventral cochlear nucleus, posterior part; VLTg, Ventrolateral tegmental area; VP, Ventral pallidum; VTA, Ventral tegmental area; X, Nucleus X.

In Figures 2A,B, distribution pattern of CB1 receptors in the brain of GASH/Sal is shown.

**Figure 3:**
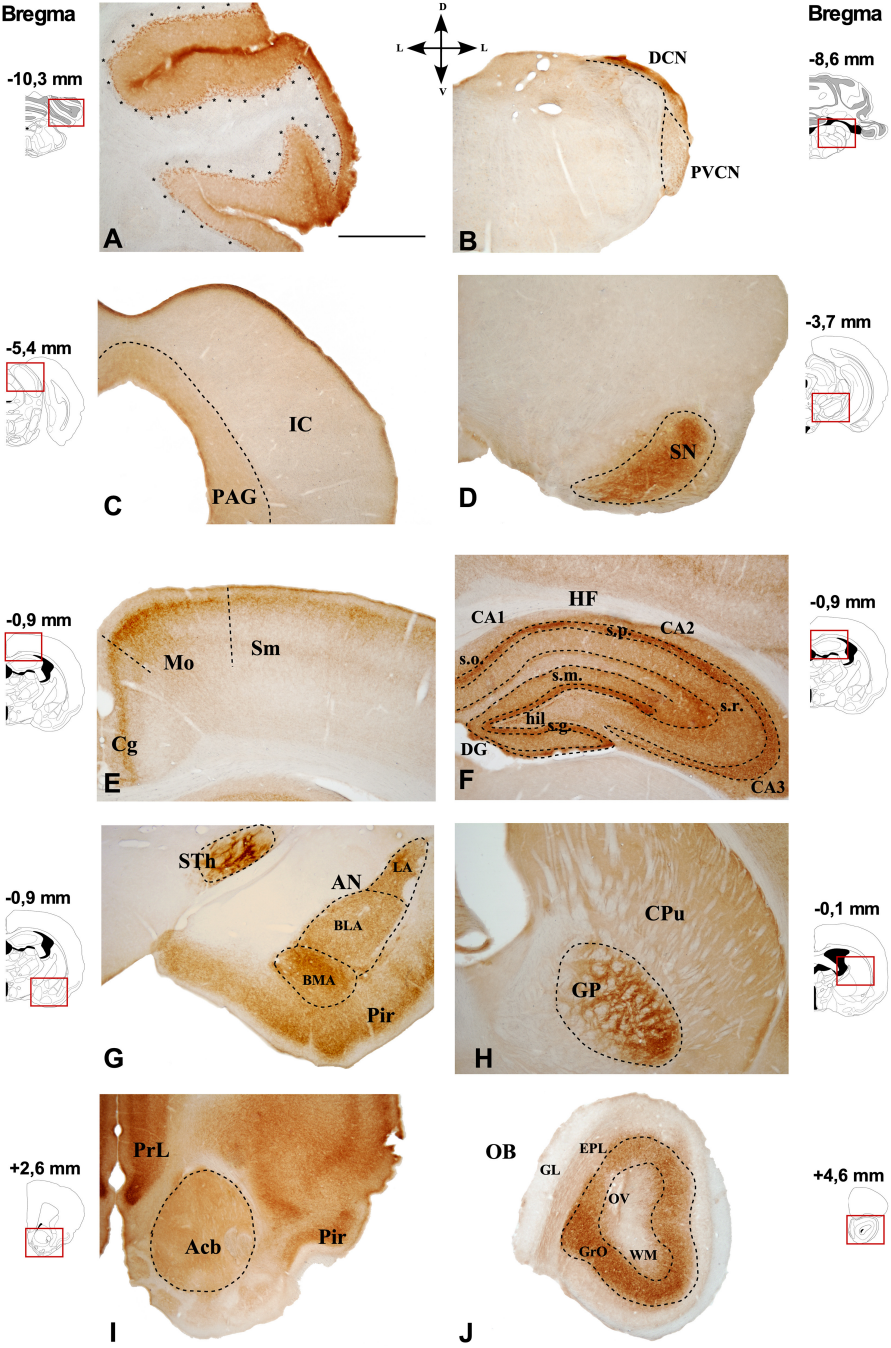
Immunostaining of CB1R in the GASH/Sal brain. Photomicrographs of GASH/Sal coronal sections referencing their rostrocaudal position as a function of Bregma and indicating its dorsoventral orientation. **(A)** Cerebellar lobe. **(B)** Dorsal and posteroventral cochlear nucleus. **(C)** Periaqueductal gray matter (delimited by dots) and the inferior colliculus. **(D)** Substantia nigra. **(E)** Somatosensory and motor cortices. **(F)** Hippocampal formation. **(G)** Piriform cortex, amygdaloid nuclei, and subthalamic nucleus. **(H)** Caudate putamen and globus pallidus. **(I)** Accumbens nucleus. **(J)** Olfactory bulb. Scale bar = 1 mm. Acb, Accumbens nucleus; AN, Amygdaloid nuclei; CA1–3, Cornu Ammonis area 1–3; BLA, Basolateral amygdala nucleus; BMA, Basomedial amygdala nucleus;Cg, Cingulate cortex; CPu, Caudate putamen; DG, Dentate gyrus; DCN, Dorsal cochlear nucleus; EPL, External plexiform layer olfactory bulb; GL, Glomerular layer olfactory bulb; GrO, Granule cell layer olfactory bulb. GP, Globus pallidus; hil., Hilus; HF, Hippocampal formation; IC, Inferior colliculus; LA, Lateral amygdala nucleus; Mo, Motor cortex; OB, Olfactory bulb; OV, Olfactory ventricle; PAG, Periaqueductal gray matter; Pir, Piriform cortex; PrL, Prelimbic cortex; PVCN, Ventral cochlear nucleus, posterior part; s.g., Stratum granulosum; s.m., Stratum moleculare; Sm, Somatosensorial cortex; SN, Substantia nigra; s.o., Stratum orients; s.p., Stratum pyramidale; s.r., Stratum radiatum; STh, Subthalamic nucleus; WM, White matter.

**Figure 4:**
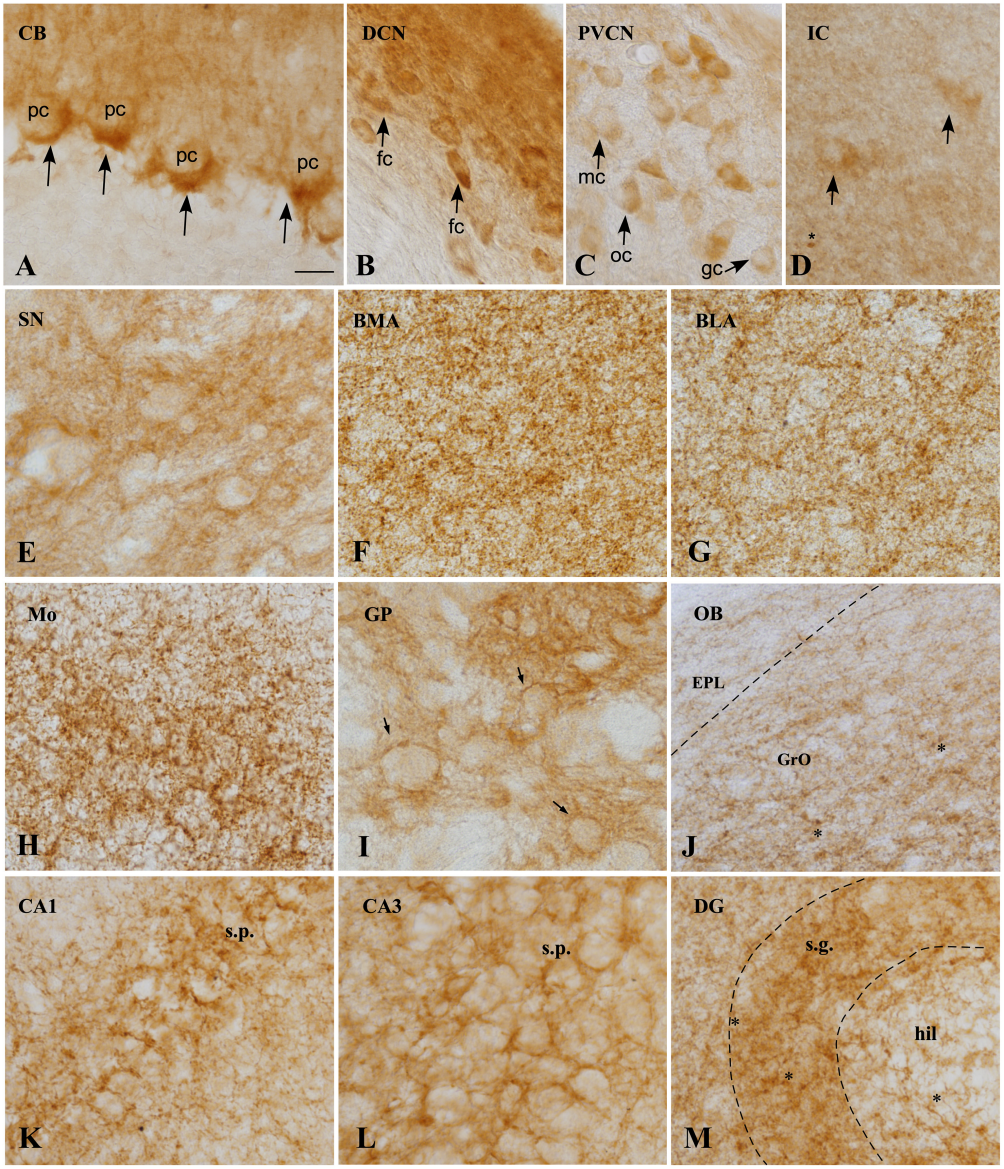
CB1 immunoreactivity in the GASH/Sal brain**. (A)** Micrographs showing CB1 receptors (arrows), around the soma and the initial part of the axon of unstained Purkinje cells, constituting the so-called “Pinceaux” formation. **(B)** Strong and diffuse CB1 immunoreactivity in the dorsal cochear nucleus. Neurons of this nucleus appear immunostained. **(C)** Neurons of the posteroventral cochlear nucleus showing slight immunoreactivity for CB1R. **(D)** CB1 expression in the central nucleus of the inferior colliculos, exhibiting diffuse immunoreactivity. Scarce medium-size neurons appears labeled intracelularlly. Asterix indicate small immunoreactive glial cells. **(E)** CB1 immunoreactivity is observed in not strong delineate fibers in the substantia nigra. **(F,G)** CB1R immunostaining in the basomedial (BML) and basolateral (BLA) amygdala, showing the neuropil granular/reticular staining. Labeling is slightly weaker in BLA. **(H)** Strong CB1 immunoreactive fibers with a plexiform pattern in the motor cortex. **(I)** High CB1 expression in the globus pallidus, where a strong network of immunoreactive fibers surround immunonegative-traversing fascicles (arrows). **(J)** CB1 immunoreactivity of the Olfactory bulb, exhibiting moderate immunoreactivity of Granule cell layer (GrO) and weakly immunoreactivity of the external plexiform layer (EPL). Asterisk indicates small immunoreactive glial cells. **(K–M)**. CB1 expression in rat hippocampal formation. CB1 positive fibers surround the somata of pyramidal cells in CA1 **(K)** and CA3 **(L)** fields of the hippocampus. Numerous varicosities, corresponding to terminals is apparent. Receptor levels are particularly high in the granule cell layer (sratum granulosum) of the dentate gyrus. Scale bar = 20 μm for all panels. BLA, Basolateral amygdala nucleus; BMA, Basomedial amygdala nucleus; CA1–3, Cornu Ammonis area 1–3 CB, Cerebellum; DCN, Dorsal cochlear nucleus, DG, Dentate gyrus; fc, Fusiform cells; EPL, External plexiform layer olfactory bulb gc, Globular cells; GP, Globus pallidus; GrO, Granule cell layer olfactory bulb; hil, Hilus; IC, Inferior colliculus; mc, Multipolar cells; Mo, Motor cortex; OB, Olfactory bulb; oc, Octopus cells; pc, Purkinje cells; PVCN, Ventral cochlear nucleus, posterior part; SN, Substantia nigra; s.g., Stratum granulosum; s.p., Stratum pyramidale.

Representative images of the CB1R immunoreactivity in the GASH/Sal are shown in the Figures 3, 4.

**Figure 5:**
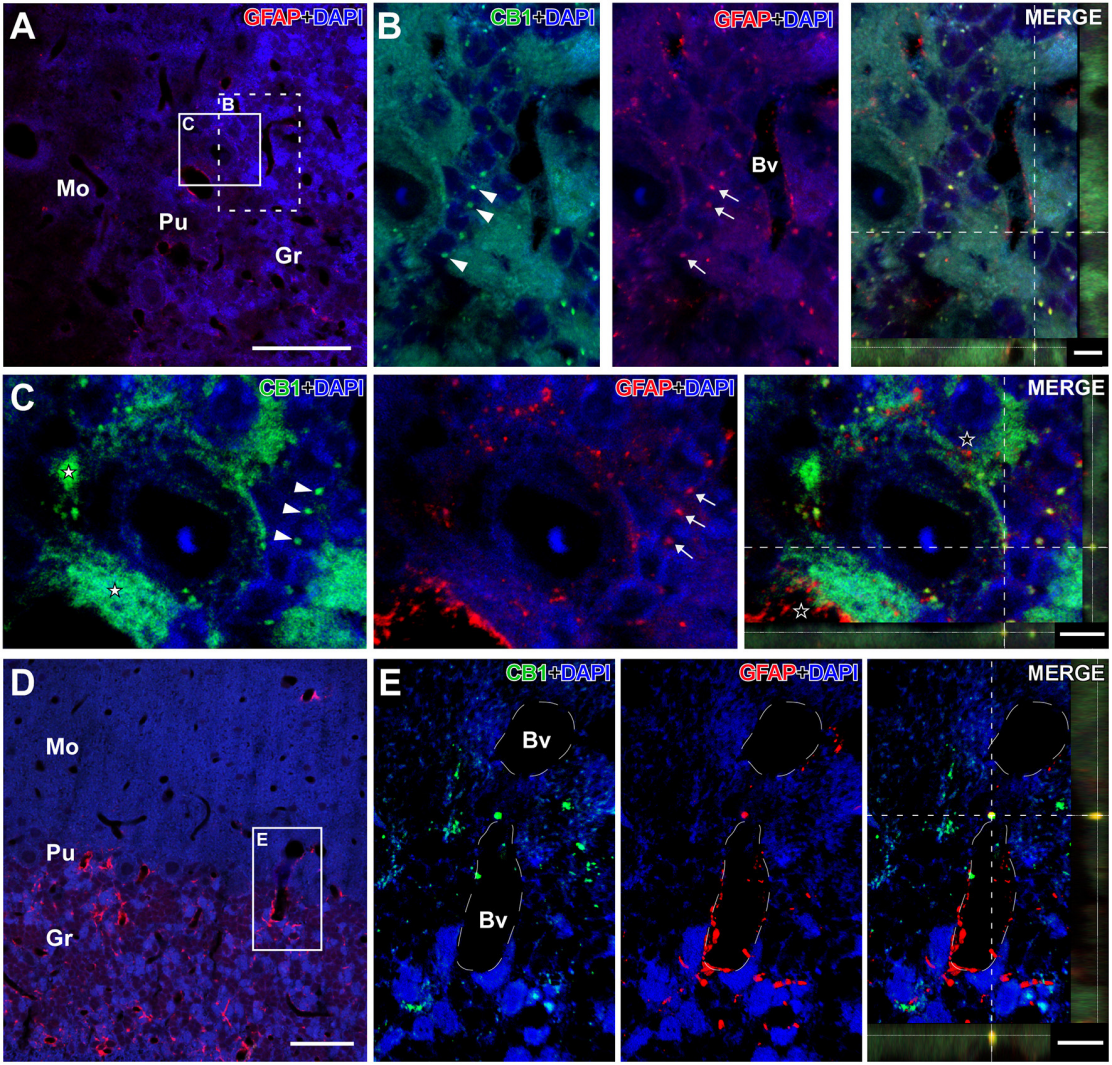
Details of CB1- and GFAP-immunolabeling in the cerebellum of the GASH/Sal. Details of CB1- and GFAP-immunolabeling in the cerebellum of the GASH/Sal (depicted in green and red, respectively). **(A)** Low magnification confocal microscopy image of a 6- μm coronal section shows immunolabeling for GFAP-immunolabeling (in red) in the cerebellum of a control hamster. **(B)** High magnification photomicrographs corresponding to the dashed square in **(A)** shows details of CB1-immunolabeled puncta (arrowheads) as well as GFAP-immunolabeled glial fibers (arrows) distributed around cerebellar granule cells. **(C)** High magnification confocal microscopy images corresponding to the white square in **(A)** shows large putative axonal puncta immunolabeled for CB1 (white stars) nearby a Purkinje cell and small CB1-immunolabeling punctate (arrowheads) in close apposition to granular cells. Note that GFAP-immunolabeled glial fibers distributed around cerebellar granule cells (arrows) as well as in the vicinity of CB1-immunolabeled terminals (black stars in the merge panel). The maximum projection of confocal images corresponding to the panels in **(C)** was displayed in the 3D video of Supplementary Material 4. **(D)** Low magnification confocal microscopy image show GFAP-immunolabeling (in red) associated with blood vessels in the cerebellum. **(E)** High magnification photomicrograph corresponding to the square in D shows details of CB1- and GFAP-immunolabeling in the vicinity of a blood vessel. Colocalization of CB1 with GFAP can be observed in the orthogonal view of the merged confocal images. DAPI (in blue) was used for nuclear staining to show cell position. Scale bars = 50 μm in **(A,D)**; 5 μm for all panels in **(B,C)**; 10 μm for all panels in **(E)**. Bv, Lumen of blood vessel; Gr, Cerebellar granular layer; Mo, Cerebellar molecular layer; Pu, Purkinje cell layer.

In general, CB1R immunostaining were found in brain microvessels throughout the brain (Figure 5D). Furthermore, labeling is seen in small cells (asterisk), presumably microglia, both in the brain stem nuclei (Figure 4D), and in the olfactory bulb (Figure 4J), cortex and hippocampus (Figure 4M).

In the cerebellum, CB1R-immunostaining was intensively present in a punctate form that were densely distributed in the cerebellar cortex, particularly in the cerebellar granular and Purkinje cell layers (Figures 3A, 4A, 5; Supplemental Materials 3, 4). The neuropil within the granule cell layer of the cerebellar cortex displayed densely CB1R-immunolabeled puncta that colabeled with GFAP (Figures 5A, 5B; Supplemental Material 3). CBR1-immunolabeled puncta of varying size were further densely seen in vicinity of Purkinje cells (Figures 4A, 5C; Supplemental Materials 3, 4). Interestingly, the large CB1R-immunolabeled puncta distributed around the soma and the initial axonal segment of the Purkinje cells (Figures 4A, 5C; Supplemental Materials 3, 4), giving rise to an arrangement described as “Pinceaux formation” (Suárez et al., 2008) that were flanked by GFAP-immunolabeled glial fibers (Figure 5C; Supplemental Materials 3, 4). CB1R- and GFAP-immunolabeling was also frequently found in the vicinity of blood vessels of the cerebellar cortex (Figures 5D, 5E).

**Figure 6:**
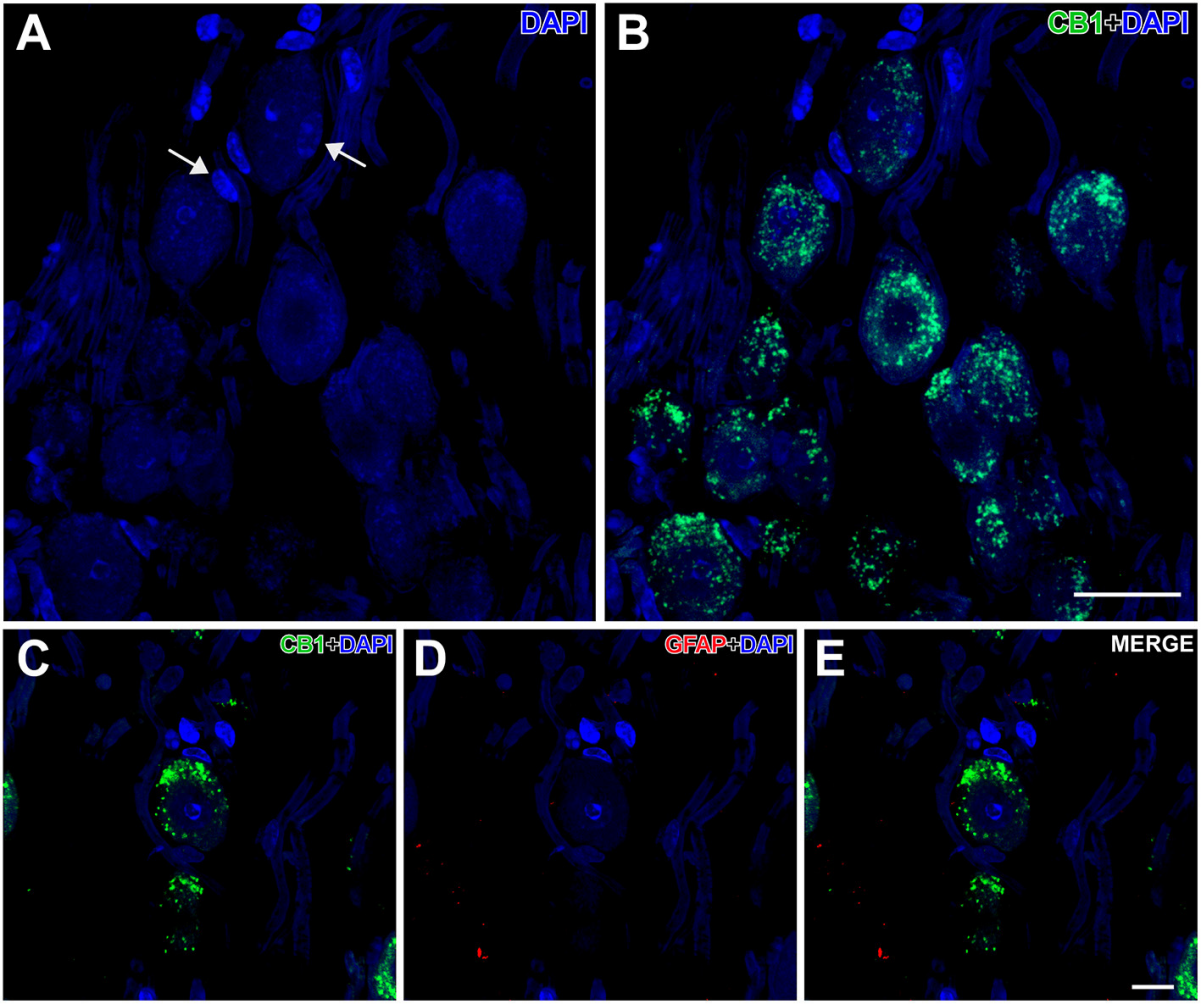
Details of CB1- and GFAP-immunolabeling in the spiral cochlear ganglion of the GASH/Sal. **(A,B)** Low magnification confocal microscopy images show spiral ganglion neurons stained with DAPI (in blue) and CB1-immunolabeling (in green) in the spiral cochlear ganglion. Note that the perikaryon of the spiral ganglion neuron is enveloped by satellite cells (arrows). **(C–E)** High magnification confocal microscopy images show CB1-immunolabeling in the cell body of a spiral ganglion neuron. Weak GFAP-immunolabeling was observed in the spiral cochlear ganglion. Scale bars = 20 μm in **(A,B)**; 10 μm in **(C–E)**.

Nuclei more directly involved in the genesis of seizures, such as the auditory nuclei (ganglion cells, cochlear nuclei, or the inferior colliculus) (Figures 4B,C) or the brainstem reticular formation (data not shown), showed CB1R immunoreactivity as well. In ganglion cells (Figure 6), CB1R immunoreactivity was distributed in the cytoplasm. In the cochlear nuclei, most of the main neurons appear immunostained in all their divisions, being more intense in the dorsal cochlear nucleus. Interestingly, this labeling is intracytoplasmic.

In the brainstem, there is slight immunoreactivity, with a diffuse staining pattern. It should be noted that structures such as the periaqueductal gray matter presented a slightly more intense marking than the adjacent inferior colliculus (Figure 3C), in which diffuse marking continues, although few immunoreactive neurons are visualized (Figure 4D).

In the substantia nigra pars reticulata (Figure 3D), dense CB1R immunoreactivity appears as fine dots or puncta (Figure 4E).

Rostrally, in the cingulate cortex (Cg), intensely stained plexus of fibers were found in the superficial layer (Figure 3E), extending to the motor (Mo) and somatosensory (Sm) cortices and, and the same plexiform staining (Figure 4H) is weaker in deeper layers. The hippocampus (Figure 3F) is distinctively immunoreactive in the principle cell layers. In both the CA1, CA2, and CA3, the stratum pyramidale (s.p.) exhibit the strongest immunostaining, with the distribution being very similar in all of them (Figures 4K,L). The intensity of the labeling in the stratum radiatum (s.r.) increase laterally. In the dentate gyrus (DG), CBR1 signal is found mainly in the granular layer (s.g.) with strong immunostaining (Figure 4M). Also, in the hilus (hil) the staining is weaker than in the pyramidal cell layer.

Larger immunoreactive fiber bundles are observed in the subthalamic nucleus (STh), as they approach the globus pallidus. In the amygdala complex (AN) (Figure 3G), an intense plexiform immunostaining is observed in all its areas, being more intense in the basomedial amygdala nucleus (Figure 4F).

The globus pallidus (GP) exhibit a strong network of fibers (reticular staining) surrounding the immunonegative traversing fascicles (Figures 1B, 3H, 4I), and the caudate putamen (Cpu) also exhibits diffuse CB1R immunoreactivity in the bundles of fibers that target the GP (Figure 3H).

Strong diffuse immunostaining is found in the accumbens nucleus (Figure 3I).

CB1Rs are also present in glomeruli of the main olfactory bulb (OB), robustly expressed in the granular layer (Figures 3J, 4J) whereas a weakly immunostained fiber plexus is found in the external plexiform layer of the olfactory bulb (EPL) and in the white matter (WM). The density of labeling in this structure is lower than that observed in the different areas of the cerebral cortex.

### Differential Gene Expression Analysis of *Cb1r* in the Brain of GASH/Sal and Control Hamsters

In control hamster brains, a uniform distribution of CB1R-immunolabeling pattern was observed across all the brain areas mentioned above. The immunostaining pattern was the same in the two hamster lines used, although some areas appeared with a slight difference in the intensity of the immunostaining.

**Figure 7:**
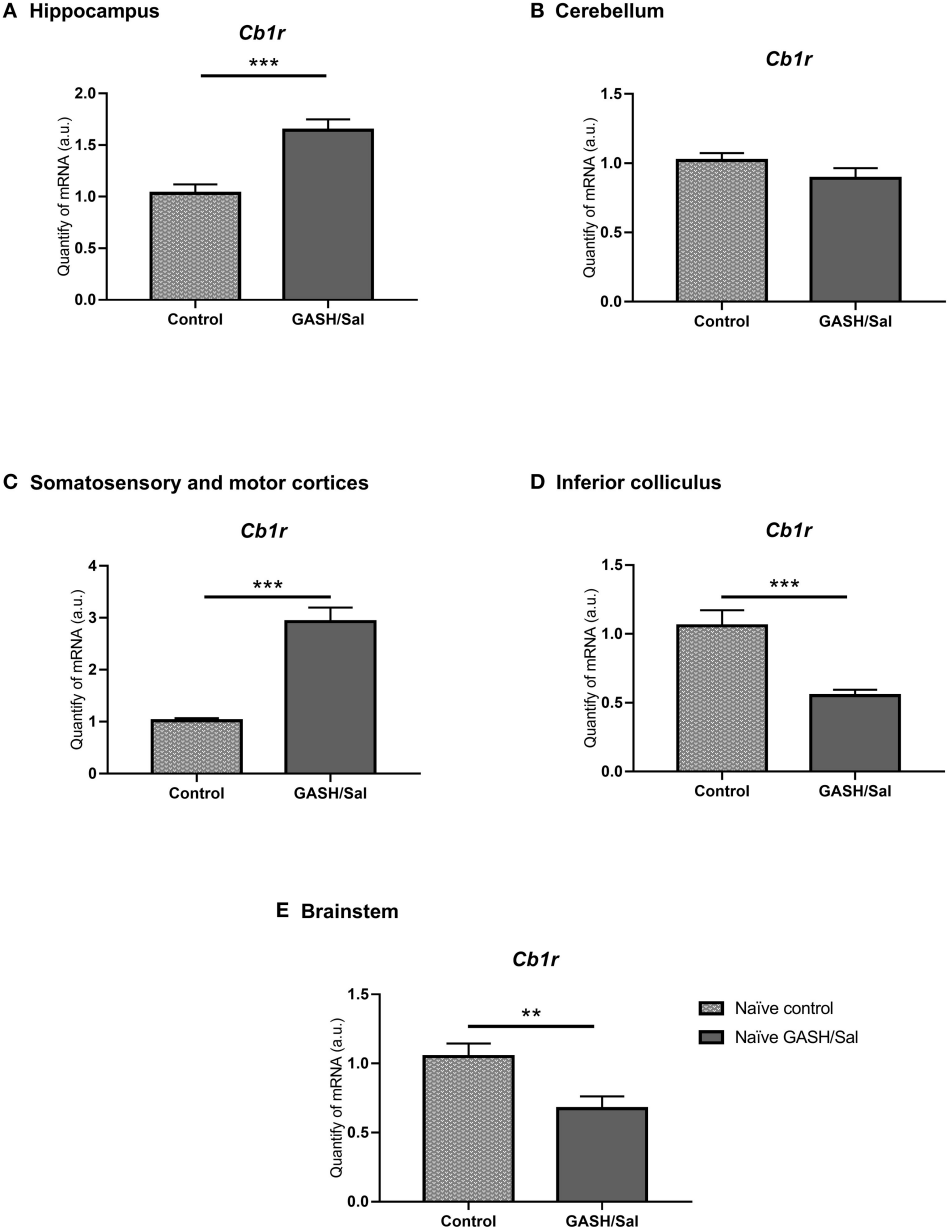
Differences in CB1R mRNA expression levels between GASH/Sal model and control. Relative quantities of transcripts in different areas of the central nervous system of the Syrian golden hamster and the GASH/Sal. In the graph, X-axis: Relative quantities of mRNA in arbitrary units; Y-axis: Experimental groups: naïve Syrian hamster (control); naïve audiogenic group (GASH/Sal). **(A)** Hippocampus; **(B)** Cerebellum; **(C)** Motor and somatosensorial cortices; **(D)** Inferior colliculus; **(E)** Brainstem Bars represent mean ± SEM. Statistical analyses: Unpaired *t*-test. ^*^p ≤ 0.01; ^**^p ≤ 0.001, and ^***^p ≤ 0.0001.

To confirm this, differential gene expression analysis of CB1R gene (*Cb1r*) was carried out in brain structures of control and GASH/Sal animals under seizure-free conditions (naïve animals) (Figure 7). These structures included the inferior colliculus (IC) (epileptogenic focus in the audiogenic strain), the hippocampus, the cerebellum, the motor and somatosensory cortices, and the brainstem. As shown in Figure 7, the RT-qPCR analysis in the IC showed significantly lower expression (^***^p < 0.0001) of the *Cb1r* in GASH/Sal animals than in the control. On the other hand, as in immunohistochemical studies, lower levels of *Cb1r* expression were detected in the cerebellum of GASH/Sal hamsters compared to controls, although this decrease is not significant in RT-qPCR analyses. For the motor and somatosensory cortices and the hippocampus, there was an increase in *Cb1r* expression in the GASH/Sal in both cases compared to controls (^***^p < 0.0001). Finally, expression levels of *Cb1r* in the brainstem were significantly lower in naïve GASH/Sal compared to naïve Syrian control hamsters (^**^ *p* < 0.01). The raw data of RT-qPCR used for analyses are included in Supplementary Material 2.

## Discussion

### CB1-Mediated Neuromodulation in Epilepsy

Although there are two types of endocannabinoid receptors, CB1R and CB2R, it is the former that is expressed in greater proportion in the central nervous system (Rosenberg et al., 2017). In fact, it is one of the most widely expressed G-protein-coupled receptors in the brain (Herkenham et al., 1991a). The endocannabinoid system acts as a retrograde control mechanism for excessive presynaptic neuronal activity (Lutz, 2004). When excessive presynaptic activity is detected, endocannabinoids are secreted from the postsynaptic terminals, bind to the CB1R of the presynaptic terminals and activate signaling cascades to decrease the liberation of neurotransmitters (Freund et al., 2003). The effects of CB1Rs depend on their location, i.e., increased CB1R signaling on glutamatergic terminals induces inhibition and neuroprotective effects, while those located on GABAergic terminals induce excitatory effects (Chiarlone et al., 2014; Guggenhuber et al., 2015).

The direct relationship between CB1R and the development of seizures in animal epilepsy models is well-documented (Lazarini-Lopes et al., 2020a). CB1R agonists exert anticonvulsant effects (Shafaroodi et al., 2004; Tutka et al., 2018), whereas CB1R antagonists block its anticonvulsant action (Wallace et al., 2002) and potentiate seizure duration and frequency (Muccioli and Lambert, 2005), suggesting that endocannabinoids might be suppressing seizure activity (Wallace et al., 2003). This hypothesis is reinforced by the fact that the activation of CB1 receptors protects against acute clonic and generalized tonic-clonic seizures in the pentylenetetrazole model (Bahremand et al., 2008). Moreover, in experiments where CB1R is blocked, audiogenic seizures become more severe (Vinogradova et al., 2011). Therefore, knowing the exact location of CB1R in animal models of epilepsy turns out to be essential to search for drugs which would enhance endocannabinoid signaling and thus modulate seizures.

In the genetically audiogenic seizure-prone hamster GASH/Sal, CB1R is distributed throughout the central nervous system. This receptor is also located in the peripheral, specifically in the spiral ganglion cells of the organ of Corti, as previously described in birds (Stincic and Hyson, 2011) and mice (Toal et al., 2016).

### Distribution of CB1R in the GASH/Sal

Neither the reactivity of the CB1R antibody used in our study nor the distribution of CB1R-immunolabeling in the cochlear has been previously tested in the brain hamster. Our study provides several evidences that indicate this CB1R antibody can be used as a marker of CB1R in brain tissue of the golden hamster as efficiently as reported in another mammal species (Fukudome et al., 2004; Rivera et al., 2014; Puighermanal et al., 2017). First, the multiple sequence alignment showed that the specific target epitope is highly conserved for CB1R in the golden hamster. Second, the pattern of CB1R-immunolabeling in the cerebellar cortex of the hamster was consistent with that described in other rodent species (Herkenham et al., 1991b; Matsuda et al., 1993; Egertová and Elphick, 2000). Furthermore, the CB1R-immunolabeled pattern in our experiments was consistently obtained using different cutting or immunodetection methods. Finally, the western blot analysis confirmed the specificity and selectivity of the CB1R antibody verifying the antibody's ability to recognize and bind to its target antigen (Supplementary Material 5).

#### Auditory Nuclei and Periaqueductal Gray Matter

In the peripheral auditory system, CB1R was located inside the cell bodies. By using specific presynaptic labeling antibodies, Stincic and Hyson (2011) showed that CB1R is in the presynaptic neuron in the chick spiral ganglion cells. Discrepancies in the location of CB1R in ganglion cells may be due to differences between birds and mammals, or due to the presence of CB1R in the cellular endosomes at steady state (Thibault et al., 2013).

The activation of endocannabinoids in the spiral ganglion has been associated to a protective effect, helping to maintain consistent response amplitudes across a long duration stimulus (Stincic and Hyson, 2011). On the other hand, CB1 receptor knockout mice possess poorer hearing thresholds than wild-type mice (Toal et al., 2016). The GASH/Sal has been described to exhibit a significant loss of spiral ganglion neurons (Sánchez-Benito et al., 2017), which results in a reduction in the amount of CB1Rs in the spiral ganglion, consistent with the significant hearing deficit in this model (Muñoz et al., 2017).

The strongest immunoreactivity for CB1R in both dorsal and ventral cochlear nuclei, has been described in the cytoplasm of main cells using autoradiographic (Herkenham et al., 1991a) and immunohistochemistry approaches (Zheng et al., 2007; Zhao et al., 2009). Zheng et al. (2007) showed the spatial distribution of CB1R in the cochlear nucleus. In that study, substantial labeling was found on many different cell types, such as stellate cells, giant cells, fusiform cells, and corn cells in the DCN, as well as globular bushy cells, elongated cells, and octopus cells in the VCN. The cytoplasmic labeling found in these cells appeared inconsistent with the reported presynaptic localization of CB1 receptors, with almost no exceptions in adult animals (Schlicker and Kathmann, 2001); however, it has since been reported that the CB1 receptor undergoes extensive trafficking between the cytoplasm and the presynaptic terminals in brain regions where it is very active (Mikasova et al., 2008). Using electron microscopy, the synaptic location of these receptors in the cochlear nucleus was confirmed (Tzounopoulos et al., 2007), in both GABAergic and glycinergic terminals, but not at auditory nerve inputs (Zhao and Tzounopoulos, 2011; Zhao et al., 2011).

The inferior colliculus (IC) is critical in audiogenic seizures (AGS) initiation (Garcia-Cairasco et al., 2017; Muñoz et al., 2017). Given the involvement of CB1R in seizures, a higher density of this receptor would be expected to be observed in the GASH/Sal' IC. However, a low expression of CB1R has been described in the IC of the GASH/Sal, the same thing that happens in other rodents (Moldrich and Wenger, 2000; Gerdeman and Lovinger, 2001). The activation of cannabinoid system in the IC through CB1 receptors can influence both GABAergic and glutamatergic neurons and exert a role in the modulation of motor behavior (Medeiros et al., 2016; Santos et al., 2020).

In summary, the presence of CB1R throughout the auditory system suggests that they play a major role in synaptic regulation (Gerdeman and Lovinger, 2001), though studies examining how activation of cannabinoid receptors affect the function of the auditory system and how CB1R expression changes after triggering seizures are needed.

Furthermore, the existence of moderate levels of CB1 receptors found in the periaqueductal gray (PAG) midbrain has been widely reported by various authors in rodents (Tsou et al., 1998; Azad et al., 2001). It has been described that, in this structure, the endocannabinoids are involved in the control of pain sensation, including stress-induced analgesia (Walker et al., 1999; Hohmann et al., 2005).

#### Cerebellum

CB1R location in GASH/Sal cerebellum, surrounding Purkinje cells (PC) had already been described (Herkenham et al., 1991a; Mailleux and Vanderhaeghen, 1992; Suárez et al., 2008). It is a typical arrangement in most rodents, though not in primates, where CB1R is found inside PCs, being postulated that these may be the substrates for the effects of cannabinoids on movement co-ordination (Ong and Mackie, 1999).

In the underlying granule cell layer, the unstained cellular bodies surrounded by scattered labeled puncta of CBR1 found in the GASH/Sal is similar to the pattern already described both in rodents (Egertová and Elphick, 2000; Suárez et al., 2008) and primates (Ong and Mackie, 1999). In our material, positive GFAP marking has also been seen in the cerebellum. It is known that in the cerebellar cortex of adult mammals there are glial cells, astrocytes and oligodendrocytes, which are classified according to their morphology (Araujo et al., 2019). In the GASH/Sal we also found CB1R and GFAP immunoreactive colocalization in some terminals of the granular layer, which could correspond to astrocytes (velate astrocytes) that are located both in the granular layer and surrounding the blood vessels (Schachner et al., 1977; Farmer and Murai, 2017).

Thus, CB1 receptors are found on virtually the main glutamate and GABA inputs to cerebellar Purkinje cells, and cannabinoids may modulate GABAergic output of the Purkinje cells PCs, therefore modulating the ongoing movement and finely regulate them.

#### Hippocampal Formation

In the GASH/Sal hippocampal formation, the main cell layers are distinctively immunoreactive. Early autoradiographic studies already showed high levels of CB1 and this hippocampal CB1R distribution pattern (Herkenham et al., 1991a; Jansen et al., 1992), confirmed in multiple studies in rodents (Kishida et al., 1980; Dove Pettit et al., 1998; Moldrich and Wenger, 2000), primates (Ong and Mackie, 1999), and birds (Stincic and Hyson, 2011). In the hippocampus, CB1 is selectively located in GABAergic axons (Katona et al., 1999). CB1 agonists have been reported to decrease the release of GABA and glutamate at hippocampal synapses, interfering with the phenomenon of long-term potentiation, which is consistent with the increased long-term potentiation observed in the CB1 knockout mice (Bohme et al., 2000). Further, rimonabant (which specifically blocks CB1Es) was shown to improve memory in rodents (Terranova et al., 1995). These data suggest that CB1R stimulation inhibits the mechanisms by which short-term memorization occurs, and its abundance in the hippocampus is related to its effects on memory processes and also makes the hippocampal formation particularly sensitive to chronic treatment with cannabinoids (Escobar Toledo et al., 2009).

#### Amygdala and Olfactory Bulb

The olfactory bulb and the amygdala are fundamental in behavior and receive highly processed sensory information. There are multiple published data about the presence of CB1R in the amygdaloid complex (AC), mainly in their cortical component, the basolateral, lateral, and basomedial nuclei (Kishida et al., 1980; Gulyas et al., 2004; Svízenská et al., 2008; Yoshida et al., 2011). In contrast to the cortical component of the amygdala, the striatal component of the AC (e.g., central and medial nuclei) displays much lower levels of CB1 receptors (Marsicano and Lutz, 1999). In our material, a moderate plexiform marking appears in their cortical component, being more intense in the basomedial nuclei. Although in the GASH/Sal we did not observe immunolabeled neurons in the AC, other authors describe moderately stained neurons in the amygdala (Tsou et al., 1998). CB1 receptors are primarily found on GABA neurons of the amygdala (Katona et al., 2001; Yoshida et al., 2011), and functional studies suggest that CB1 receptors and endocannabinoids facilitated extinction of fear conditioning via inhibiting GABA release in this area (Marsicano et al., 2002).

Regarding the olfactory bulb, there are important differences in CB1R labeling according to phylogeny. The immunostaining pattern in GASH/Sal is very similar to that found in other rodents, such as rats and mice, being the granular cell layer (GrO) the one that presents the highest amount of CB1R, followed by the inner plexiform layer, while less is expressed in the external plexiform layer (EPL) and the glomerular layer (GL) (Herkenham et al., 1991b; Tsou et al., 1998; Egertová and Elphick, 2000). In the glomerular layer (GL), there is slightly more CB1R labeling in rat and mice that in the GASH/Sal. Interestingly, no CB1R immunoreactivity was observed in the EPL in mice (Soria-Gómez et al., 2014). Other mammals, such as the dog, exhibit a labeling pattern in the OB different from rodents, being not only intense in the glomerular layer, but also in the granular layer (Freundt-Revilla et al., 2017). There are also differences with men, since CB1R is not expressed in the olfactory bulb or in the olfactory epithelium (Lötsch and Hummel, 2015).

In rodents, CB1R have been reported/described to be abundantly expressed on axon terminals of centrifugal cortical glutamatergic neurons that project to inhibitory granule cells of the main olfactory bulb (MOB) and seem to be associated with the odor detection increasing, promoting food intake (Soria-Gómez et al., 2014).

#### Basal Ganglia: Globus Pallidus, Substantia Nigra, and Caudate Putamen

In the GASH/Sal, as in most rodents, CB1 receptor levels in the basal ganglia are among the highest in the entire nervous system, and within these structures, the GP and SN present the highest expression (Herkenham et al., 1991b; Egertová and Elphick, 2000). These receptors are in fibers that surround immunonegative neurons (Egertová and Elphick, 2000; Egertová et al., 2003) and GP immunonegative fascicles (Sañudo-Peña et al., 1999), that arise from incoming axonal projections from other brain regions (Matsuda et al., 1993). In the caudate and putamen, there are numerous bundles of immunoreactive fibers that target the GP. It has been described that, CB1 is found presynaptically in the neurons of this nucleus, in fibers that come from the striatum through GABAergic pathways (striatonigral and striatopalidal) (Romero et al., 2002). Also, a low but significant percentage of CB1-immunoreactivity is co-localized with tyrosine hydroxylase (TH), a marker for both noradrenergic and dopaminergic terminals (Köfalvi et al., 2005). This suggests that there is a sophisticated presynaptic regulation in the basal ganglia, involved in the initiation and execution of a movement, and its motor activity is regulated in part by CB1 receptors. This is supported by publications that describe that CB1 receptor binding was altered in the basal ganglia of humans affected by several neurological diseases (Consroe, 1998) and of rodents with experimentally induced motor disorders (Zeng et al., 1999; Romero et al., 2000). Once again, the need to investigate the possible changes in the activation of these receptors in GASH/Sal after seizures is confirmed, to see their possible role in the convulsive process.

#### Cortical Areas

We found CB1 receptors densely expressed in all regions of the GASH/Sal cortex, similar to the plexiform pattern reported in other rodents, particularly in the somatosensory, cingulate, perirhinal, entorhinal, motor, and piriform cortices (Tsou et al., 1998; Marsicano and Lutz, 1999; Egertová and Elphick, 2000; Moldrich and Wenger, 2000; Mackie, 2005). These cannabinoid receptors may have a major role in inhibiting presynaptic calcium channels, reducing release of number of neurotransmitters, which implies a role for endocannabinoids in modulating processes as important as perception, attention, and behavior, depending on the cortical zone. CB1-immunoreactivity is quite similar within primates, with small differences in the CB1R distribution in the different cortical layers, and also in the localization both pre- and post-synaptic, suggesting that the CB1R role is broader than merely mediating presynaptic inhibition (Glass et al., 1997; Ong and Mackie, 1999).

#### Non-neuronal Cells

Finally, CB1R was found in astrocytes and blood vessels. All major cell types involved in cerebrovascular control pathways (i.e., smooth muscle, endothelium, neurons, astrocytes, pericytes, microglia, and leukocytes) are capable of synthesizing endocannabinoids and/or express some or several of their target proteins, as CB1 and CB2 receptors (Galiègue et al., 1995). Therefore, the endocannabinoid system may importantly modulate the regulation of cerebral circulation under physiological and pathophysiological conditions in a very complex manner. Experimental data accumulated since the late 1990s indicate that the direct effect of cannabinoids on cerebral vessels is vasodilation mediated, at least in part, by CB1 receptors (Wagner et al., 2001).

In summary, the pattern of distribution of cannabinoid receptors in the GASH/Sal is highly similar to that described in other mammal species (Freundt-Revilla et al., 2017; Silver, 2019).

### Differential Gene Expression Analysis of CB1 Receptor in the Brain of GASH/Sal and Control Hamsters

There were some areas in the brain of control animals that showed small immunoreactivity differences compared the GASH/Sal model, such as the inferior colliculus, cerebellum, the anterior commissure or the periaqueductal gray matter (data not shown). These results were correlated with expression analysis of the gen encode the CB1R, *Cb1r*. In the caudal brainstem and the inferior colliculus, the highest levels of *Cb1r* mRNA were obtained in the control hamster. This decrease in the *Cb1r* in the GASH/Sal was detected under basal conditions, as the animals were not subjected to any acoustic stimulation and therefore did not have any seizures. The cannabinoid system has been described as having a role in the downward regulation of auditory stimuli in some neurons of the inferior colliculus (Valdés-Baizabal et al., 2017). Since CB1 receptors are known to inhibit the release of many neurotransmitters, it is therefore conceivable that a change in the number or function of CB1 receptors could alter their excitability and calcium influx. The fact that our model has a lower gene expression of *Cb1r* in this region could favor the loss of this type of intrinsic physiological control, which could be precipitating a pro-epileptogenic environment in the inferior colliculus.

Specific experiments are necessary to determine which specific neurons contain these CB1 receptors to better understand the scope of the variation of endogenous CB1R expression in the audiogenic nucleus.

No significant results were obtained when comparing *Cb1r* expression between the GASH and control cerebellum. However, we found higher expression of *Cb1r* in the motor cortex, the somatosensory cortex and the hippocampus of epileptic animals, despite not having shown differences in the immunohistochemical study (data not shown). It is well-reported that in the somatosensorial cortex, CB1Rs are expressed exclusively expressed in Cholecystokinin-positive and Calbindin-positive GABAergic interneuron axons (Bodor et al., 2005). Knowing that these cells could adjust population synchrony inhibition and the input plasticity in intracortical circuits, one may think that higher expression of these receptor and activation by endocannabinoids could enhance the depolarization-induced suppression of inhibition in these circuits, heightening their excitatory inputs and, therefore, affecting intracortical communication. In the motor cortex layers II-III, GABAergic neurons express CB1R (Marsicano and Lutz, 1999). Moreover, these receptors regulate dopamine secretion and activity (Melis et al., 2004; Laviolette and Grace, 2006), which in last term promotes the growth of pyramidal neurons in particular areas of the cortex via D1 receptors (Stanwood et al., 2005; Ballesteros-Yáñez et al., 2007). Hence, upregulation of Cb1r mRNA may have an effect in the extension of pyramidal neurons, increasing its arborization and, therefore increasing their synaptic capacity.

The increase of *Cb1r* expression in the hippocampus, and in the amygdala, has been described in other epileptic animal models (Lazarini-Lopes et al., 2020b). It has been postulated that this constitutive increase in endocannabinoids in animal models of epilepsy could have a possible neuroprotective mechanism (via decreasing excitability and synchronization by reducing glutamate and GABA release) (Guggenhuber et al., 2010; Goffin et al., 2011).

Additionally, in the Wistar audiogenic rat strain (WAR), a genetic model of audiogenic epilepsy, exhibit and endogenous increase of CB1R immunostaining in the hippocampus and amygdala after acute and chronic audiogenic seizures (Lazarini-Lopes et al., 2020b). These recent data reinforce the link between the limbic system and seizure susceptibility and provide new knowledge on the role of the endocannabinoid system in the control of neuronal excitability.

Preliminary results in our laboratory show that, after repetitive acoustic stimulation in controls and in GASH/Sal, there is an increase in *Cb1r* expression in the IC of GASH/Sal (data not shown).

An increase in the activity of the endocannabinoid system in stressful situations has been described as a mechanism to reduce anxiety (Lutz et al., 2015). The CB1R increase observed after the seizures, could be part of the physiological response of the GASH/Sal to mitigate the stress produced by the crisis. New experiments are necessary to study the changes in CB1 receptors after seizures and after the administration of cannabinoid agonists/antagonists, to see more directly the role of this cannabinoid receptor in the generation and maintenance of seizures in our epilepsy model.

## Conclusion

The endocannabinoid system is widely distributed in the central nervous system of the animals analyzed in this study. We showed the immunohistochemical and gene expression analysis of the GASH/Sal model, comparing it with control hamsters and with what has already been described in the literature.

There is a lower density of CB1R in the epileptogenic focus of the GASH/Sal model, the inferior colliculus, which could lead to hyperexcitability. However, the presence of CB1R in the peripheral auditory system indicates that the activation of endocannabinoids may also regulate the encoding of auditory information at its earliest stages in the brain, which is important due to the alterations found in the spiral ganglion of the genetically audiogenic seizure-prone hamster GASH/Sal. On the other hand, we find higher gene expression of the CB1 receptors in the motor cortex and the hippocampus, which has been related to a neuroprotective mechanism in epileptic animals. Despite these differences, we consider that the endocannabinoid system in the GASH/Sal hamster is extremely similar to that of other rodents. These results can be used as a basis for further studies aiming to better understand the pharmacological and behavioral effects associated with cannabinoid exposure.

## Data Availability Statement

The raw data supporting the conclusions of this article will be made available by the authors, without undue reservation.

## Ethics Statement

The animal study was reviewed and approved by Bioethics Committee of the University of Salamanca (approval number 380).

## Author Contributions

DL and AF-H: conceptualization and funding and supervised the study. JG: characterization of the primary antibody. LZ: experiments of RT-qPCR and the thin sections' immunostaining. RM: experiments of immunohistochemistry. RG-N: confocal analysis. AF-H and JG: original draft preparation. All authors: visualization, review and editing, and have read and agreed to the published version of the manuscript.

## Conflict of Interest

The authors declare that the research was conducted in the absence of any commercial or financial relationships that could be construed as a potential conflict of interest.

## Supplementary Material

Supplementary Material 1 Conserved sequence from CB1R identified by EBI Clustal Omega program. The table shows sequence alignment of the CB1R sequences in mouse and hamster, with only two aminoacid differences in the region 120 (green square). The epitope sequence, C-terminal 31aa (nm007726) is the same in mouse and hamster (red square).

Supplementary Material 2 Raw data of RT-qPCR used for analyses. The qPCR data included a set of six biological replicates (sample cases) for each experimental group (treatment condition), triplicate technical replicates for all structures and genes as well as the Ct values of β-actin housekeeping gene used. Undetermined replicates were not used for analysis due to Cts differences over 0.5. Undetermined data were excluded of the statistical analysis due to high differences among the Ct value among replicates. Also, some samples were discarding for the low quality of RNA. *Actb*, β-actin used as housekeeping gene; *Cb1r*, the cannabinoid receptor type 1.

Supplementary Material 3 Confocal microscopy images of the GASH/Sal cerebellum showing CB1-immunolabeled puncta (in green) distributed around unlabeled cell bodies and dendrites of Purkinje cells (asterisks) as well as GFAP-immunolabeled glial fibers (in red). Note the dense CB1-immunolabeling punctate in the cerebellar granular and Purkinje cell layers. The confocal images were taken from a 6- μm coronal section and DAPI (in blue) was used for nuclear staining to show cell position. Gr, cerebellar granular layer; Mo, cerebellar molecular layer; Pu, Purkinje cell layer. Scale bars = 10 μm for all panels.

Supplementary Material 4 3D reconstruction and 360-degree rotational rendering video of the confocal image stacks corresponding to Figure 5C shows putative axonal puncta immunolabeled for CB1 in close apposition to a cell body of a Purkinje cell and granular cells. GFAP-immunolabeling is displayed in red and DAPI staining (in blue) was used for nuclear staining to show cell position. Scale bar = 20 μm.

Supplementary Material 5 Immunoblotting. Single protein bands were detected with the rabbit anti-CB1R antibody in the Syrian hamster cerebellum, both in GASH/Sal and control hamsters, showing antibody specificity.
