# Supplementary figures and images for: Distribution of the Cannabinoid Receptor Type 1 in the Brain of the Genetically Audiogenic Seizure-Prone Hamster GASH/Sal
Source: Front Behav Neurosci. 2021 Mar 24;15:613798. doi: 10.3389/fnbeh.2021.613798 (PMC8024637; doi:10.3389/fnbeh.2021.613798)

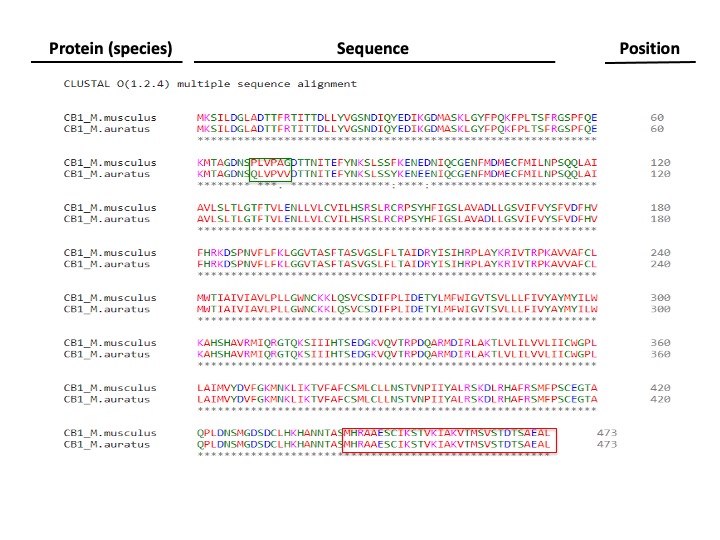

Supplement: Supplementary Material 1 — Conserved sequence from CB1R identified by EBI Clustal Omega program. The table shows sequence alignment of the CB1R sequences in mouse and hamster, with only two aminoacid differences in the region 120 (green square). The epitope sequence, C-terminal 31aa (nm007726) is the same in mouse and hamster (red square). [file Image_1.JPEG]

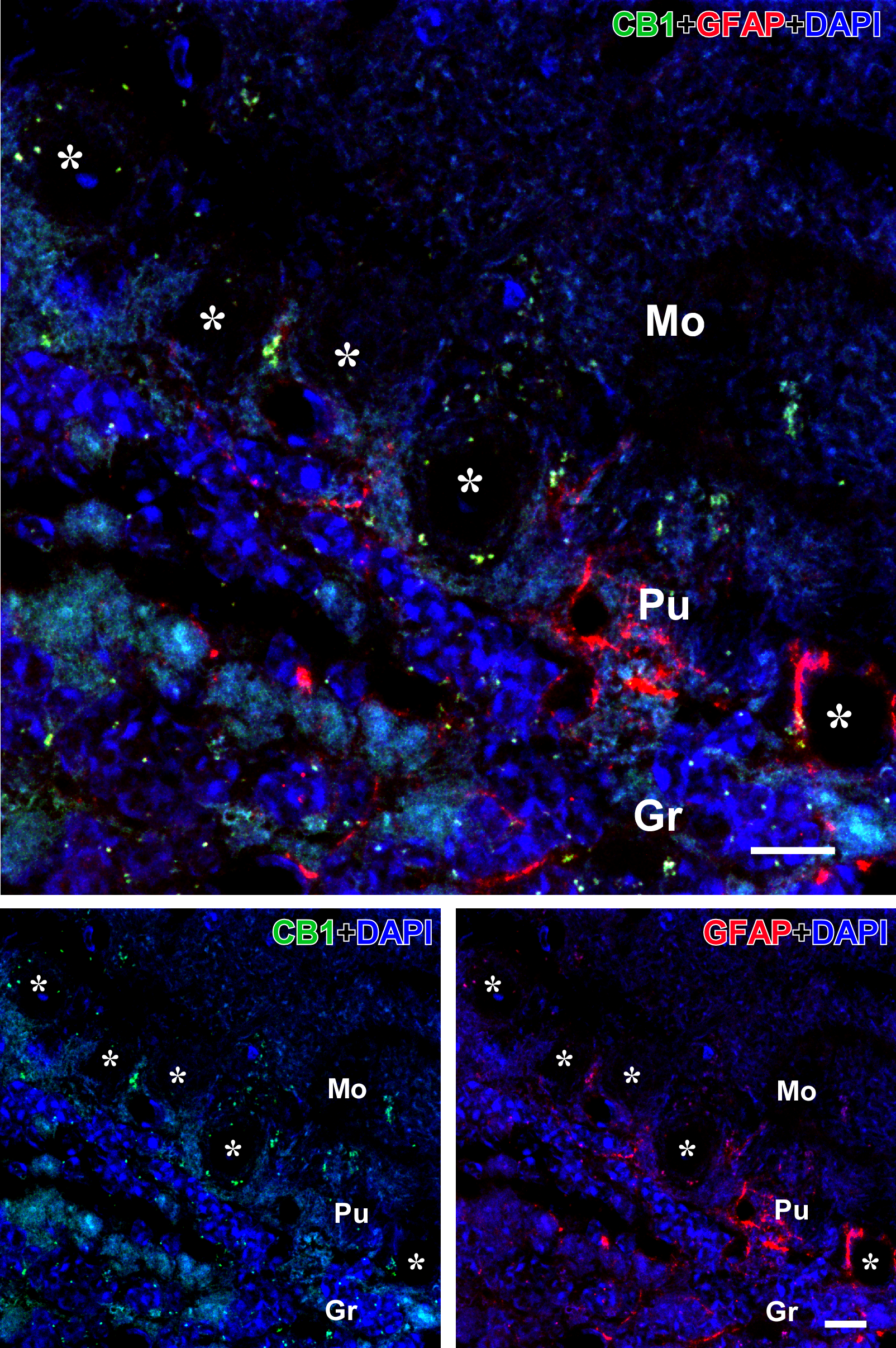

Supplement: Supplementary Material 3 — Confocal microscopy images of the GASH/Sal cerebellum showing CB1-immunolabeled puncta (in green) distributed around unlabeled cell bodies and dendrites of Purkinje cells (asterisks) as well as GFAP-immunolabeled glial fibers (in red). Note the dense CB1-immunolabeling punctate in the cerebellar granular and Purkinje cell layers. The confocal images were taken from a 6- μm coronal section and DAPI (in blue) was used for nuclear staining to show cell position. Gr, cerebellar granular layer; Mo, cerebellar molecular layer; Pu, Purkinje cell layer. Scale bars = 10 μm for all panels. [file Image_2.TIF]

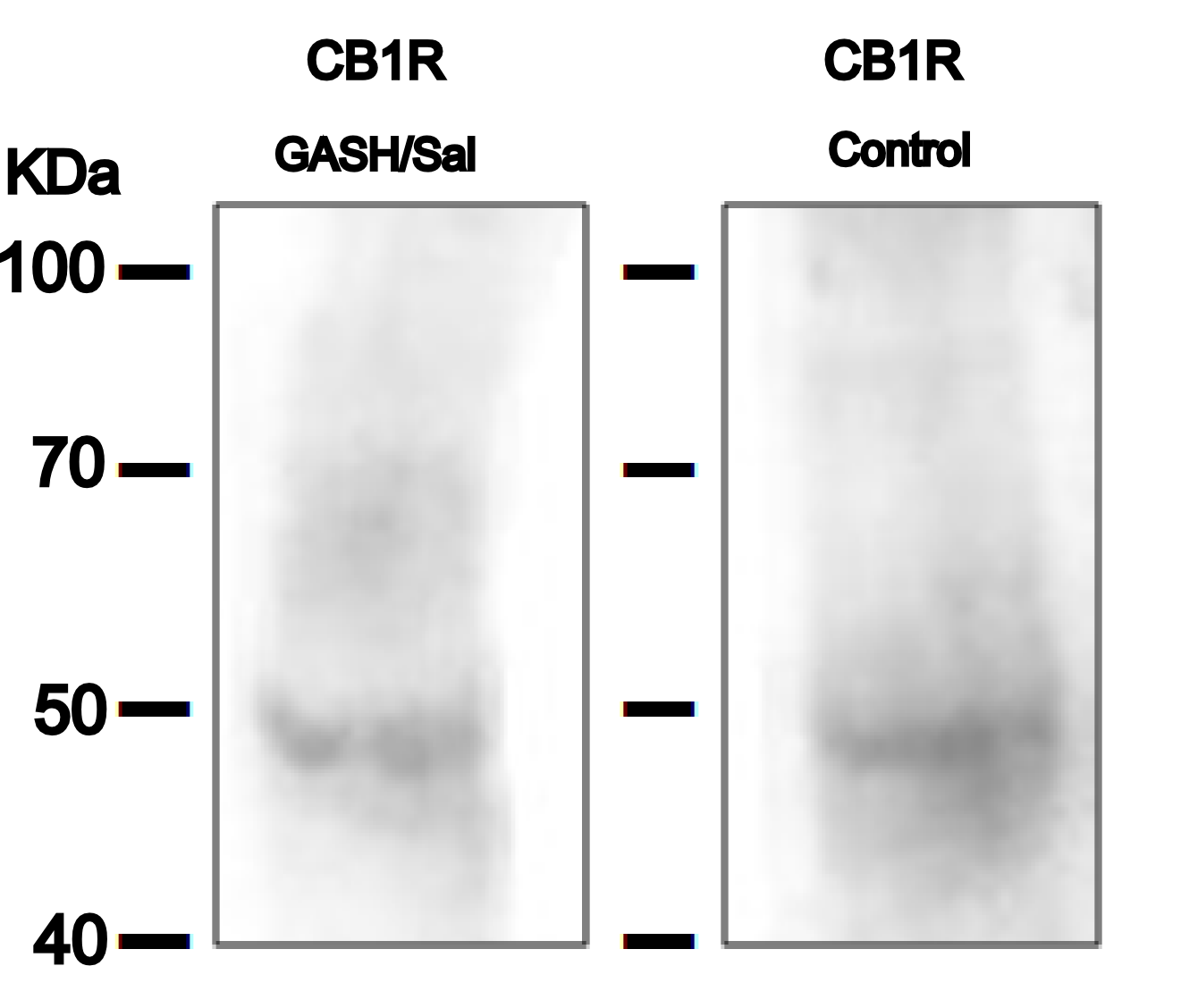

Supplement: Supplementary Material 5 — Immunoblotting. Single protein bands were detected with the rabbit anti-CB1R antibody in the Syrian hamster cerebellum, both in GASH/Sal and control hamsters, showing antibody specificity. [file Image_3.TIF]
