# Supplementary material for: Distribution of the Cannabinoid Receptor Type 1 in the Brain of the Genetically Audiogenic Seizure-Prone Hamster GASH/Sal
Source: Front Behav Neurosci. 2021 Mar 24;15:613798. doi: 10.3389/fnbeh.2021.613798 (PMC8024637; doi:10.3389/fnbeh.2021.613798)
Supplement: Supplementary Material 2 — Raw data of RT-qPCR used for analyses. The qPCR data included a set of six biological replicates (sample cases) for each experimental group (treatment condition), triplicate technical replicates for all structures and genes as well as the Ct values of β-actin housekeeping gene used. Undetermined replicates were not used for analysis due to Cts differences over 0.5. Undetermined data were excluded of the statistical analysis due to high differences among the Ct value among replicates. Also, some samples were discarding for the low quality of RNA. Actb, β-actin used as housekeeping gene; Cb1r, the cannabinoid receptor type 1. [file Data_Sheet_1.PDF]

Raw data of RT-qPCR used for analyses (Ct values)												
Animal group	Animal ID	Replicates	Hippocampus		Cerebellum		Somatosensory and motor cortex		Inferior colliculus		Brainstem	
			Cb1r	*Actb*	*Cb1r*	*Actb*	*Cb1r*	*Actb*	*Cb1r*	*Actb*	*Cb1r*	*Actb*
**NAÏVE CONTROL**	Animal 1	1	24,688	17,313	22,477	18,695	25,469	18,334	21,658	17,622	26,528	19,862
		2	24,425	17,305	22,366	18,725	25,333	18,488	21,628	17,882	Undetermined	Undetermined
		3	24,662	17,221	22,475	18,639	25,418	18,459	21,599	17,853	26,811	19,849
	Animal 2	1	25,874	18,611	21,339	17,608	27,842	20,730	21,558	16,925	24,873	17,259
		2	25,864	19,176	21,230	17,602	28,240	20,722	21,778	16,749	25,012	17,272
		3	25,701	18,457	21,267	17,576	28,144	20,742	21,734	16,776	24,963	17,198
	Animal 3	1	Undetermined	Undetermined	20,761	17,578	23,947	17,122	21,958	16,920	26,004	17,601
		2	24,052	17,146	20,717	17,292	24,276	17,442	21,892	16,980	25,706	17,657
		3	23,969	17,430	20,634	17,242	24,068	17,130	21,935	16,815	Undetermined	Undetermined
	Animal 4	1	24,997	17,451	21,998	17,754	25,092	17,668	22,136	17,603	24,213	16,543
		2	25,096	17,433	21,947	17,749	25,199	17,729	22,224	17,701	24,523	16,681
		3	25,075	17,423	21,965	17,764	25,204	17,843	22,245	17,636	24,289	16,695
	Animal 5	1					26,256	18,526			25,824	17,727
		2					26,090	18,529			25,984	17,723
		3					26,007	18,530			25,788	17,682
	Animal 6	1			20,608	16,838	26,636	19,097			25,503	17,591
		2			20,495	16,853	26,499	19,088			25,298	17,600
		3			20,553	16,850	26,488	19,127			25,396	17,641
**NAÏVE GASH/SAL**	Animal 7	1			21,944	17,862	24,589	18,478	25,680	19,874	30,617	21,912
		2			22,001	17,918	24,601	18,450	25,881	19,870	30,286	21,899
		3			21,866	17,924	24,383	18,587	25,844	19,724	30,516	21,937
	Animal 8	1	21,424	15,092			23,689	17,574	25,324	20,179	30,488	21,801
		2	21,602	15,015			24,016	17,603	25,099	20,123	31,473	21,781
		3	21,663	15,181			23,758	17,578	25,118	20,264	30,456	21,833
	Animal 9	1			25,029	20,370	24,045	18,387	24,430	19,136		
		2			24,933	20,363	24,843	18,302	24,470	19,085		
		3			24,980	20,302	24,664	18,433	24,365	19,054		
	Animal 10	1	23,400	17,082	22,989	18,953	24,003	17,947	24,622	19,093	26,574	19,184
		2	23,421	17,091	22,784	18,952	24,400	17,893	24,791	19,100	27,145	19,184
		3	23,380	17,030	22,771	18,963	Undetermined	Undetermined	24,721	19,068	Undetermined	Undetermined
	Animal 11	1	22,394	16,147	21,691	17,506	23,278	17,924			30,639	21,547
		2	22,387	16,206	21,147	17,579	23,314	18,017			30,825	21,528
		3	22,381	15,758	21,110	17,584	23,227	17,954			30,849	21,552
	Animal 12	1	22,924	16,335	21,129	17,824	23,751	18,810			26,735	19,327
		2	23,224	16,357	Undetermined	Undetermined	23,933	18,826			27,091	19,355
		3	23,862	16,210	21,265	17,965	24,020	18,880			27,366	19,315
